# Anti-Retroviral Treatment Outcomes among Older Adults in Zomba District, Malawi

**DOI:** 10.1371/journal.pone.0026546

**Published:** 2011-10-21

**Authors:** Joel Negin, Monique van Lettow, Medson Semba, Alexandra Martiniuk, Adrienne Chan, Robert G. Cumming

**Affiliations:** 1 School of Public Health, University of Sydney, Sydney, Australia; 2 Dignitas International, Zomba, Malawi; 3 Dalla Lana School of Public Health, University of Toronto, Toronto, Canada; 4 Ministry of Health, Zomba, Malawi; 5 George Institute for Global Health, Sydney, Australia; 6 Sunnybrook Health Sciences Research Institute, University of Toronto, Toronto, Canada; 7 Department of Medicine, University of Toronto, St. Michael's Hospital, Toronto, Canada; Asociacion Civil Impacta Salud y Educacion, Peru

## Abstract

**Background:**

There are approximately 3 million people aged 50 and older in sub-Saharan Africa who are HIV-positive. Despite this, little is known about the characteristics of older adults who are on treatment and their treatment outcomes.

**Methods:**

A retrospective cohort analysis was performed using routinely collected data with Malawi Ministry of Health monitoring tools from facilities providing antiretroviral therapy services in Zomba district. Patients aged 25 years and older initiated on treatment from July 2005 to June 2010 were included. Differences in survival, by age group, were determined using Kaplan–Meier survival plots and Cox proportional hazards regression models.

**Results:**

There were 10,888 patients aged 25 and older. Patients aged 50 and older (N = 1419) were more likely to be male (P<0.0001) and located in rural areas (P = 0.003) than those aged 25–49. Crude survival estimates among those aged 50–59 were not statistically different from those aged 25–49 (P = 0.925). However, survival among those aged 60 and older (N = 345) was worse (P = 0.019) than among those 25–59. In the proportional hazards model, after controlling for sex and stage at initiation, survival in those aged 50–59 did not differ significantly from those aged 25–49 (hazard ratio 1.00 (95% CI: 0.79 to 1.27; P = 0.998) but the hazard ratio was 1.46 (95% CI: 1.03 to 2.06; P = 0.032) for those aged 60 and older compared to those aged 25–49.

**Conclusions:**

Treatment outcomes of those aged 50–59 are similar to those aged 25–49. A better understanding of how older adults present for and respond to treatment is critical to improving HIV services.

## Introduction

There are approximately 3 million people aged 50 and older in sub-Saharan Africa who are HIV-positive, representing more than 13% of all HIV cases in the region [1]. These figures are likely to increase as the number of people on life-prolonging anti-retroviral treatment (ART) grows. Despite this, older people have been neglected in the global and regional HIV response. UNAIDS and other prominent data sources report prevalence rates of those aged 15–49 [2] and few services directly target those aged 50 and older [3], [4].

The evidence base on ART presentation, initiation and outcomes for older adults from developed countries has been growing over the past few years [5], [6]. There is evidence that older adults are more likely to be late presenters with more AIDS-defining diseases and are more likely to die before treatment initiation [7]–[10]. After initiation of ART, studies have also shown higher mortality [11] and slower immune system reconstitution [12] leading some to suggest that older adults should initiate treatment earlier than younger counterparts [13].

There has not however been much analysis conducted on older adults in sub-Saharan Africa [14], [15]. Recently, one Ugandan and one South African study provided some of the first evidence reporting on treatment outcomes revealing elderly age status (50 and older) associated with higher mortality [16], [17].

A recent study revealed that older Africans are less likely to have a good understanding of HIV, are less likely to have been tested and are less likely to be aware of treatment availability [18]. Along with this, for older adults in sub-Saharan Africa, issues of stigma, lack of access to health services, poor overall health [19] and ageist views on sexual behavior might negatively impact on the effectiveness of ART scale-up efforts.

In Malawi, a country with a 2009 HIV prevalence of 11% among those aged 15–49, analysis of 2007 UNAIDS data suggested that there are approximately 156,000 people over 50 who are living with HIV in Malawi or 16.8% of the total [1]. Furthermore, people over 50 make up 9.3% of Malawi's population [20]. ART coverage has expanded dramatically over the past few years and, as of 2009, 72% of those who needed treatment were receiving it based on 2006 World Health Organization ART guidelines [2] suggesting that the numbers ageing with HIV will increase. Despite this, no published work exists for Malawi specifically examining HIV among older adults.

Using data from Zomba District in Malawi, this study examines the characteristics of older adults on ART, their condition at initiation and their treatment outcomes compared against younger adults.

## Methods

Subjects were adults aged 25 and older at time of ART initiation residing in Zomba District in southern Malawi who accessed ART services in the district's 23 health facilities between 1 July 2005 and 30 June 2010.

The methods used in this study have been described in detail elsewhere (Chan et al 2010) [21]. In brief, a retrospective cohort analysis was performed using data collected with standard Malawi Ministry of Health (MOH) ART monitoring tools. ART provision was implemented as per MOH guidelines based on WHO clinical staging or CD4 count <250 cells ?mm3 where measurement of CD4 is available. The first line ART regimen in Malawi is stavudine, lamivudine and nevirapine in fixed-dosed combination. After initiation, follow-up was monthly and then, after approximately 6 months, patients were followed less often depending on assessment of adherence. In the project district, mortality was documented by trained health workers in health facilities and by reports from trained patient guardians where deaths occurred at home. Defaulter tracing was also conducted which revealed additional deaths.

Analyses in this paper only included those aged 25 and older to exclude those regarded by MDG [22] and UNGASS [23] indicators as adolescents. Age was defined as age at treatment initiation. The 23 health facilities where MOH ART provision is supported by Dignitas International were classified as urban, semi-urban or rural based on location and catchment area of facility. Analysis of conditions at initiation was based on examination at baseline of 52 HIV-related conditions detailed in WHO clinical staging including: acute ulcerative mouth infections, Kaposi's sarcoma, various types of pneumonia and weight loss to name a few. As per Malawi MOH guidelines, individuals were initiated based on characterization as being in WHO clinical stage 3 or 4 or, if characterized as Stage 1 or 2, based on low CD4 count if available. As a result, baseline CD4 counts were collected for only 47% of patients. Additionally, CD4 testing capacity was only available in urban settings with blood samples from rural patients being sent to urban facilities as needed.

Statistical analyses included Chi-square tests for categorical variables and t-tests for continuous variables to compare differences between groups. Logistic regression analyses were used for categorical outcomes including WHO clinical stage and number of conditions at initiation. Interactions between age and sex and between age and location of initiation were tested in the logistic regression analyses. Cox proportional hazard regression models were used to evaluate relationships between age, sex and status at initiation and treatment outcomes (death). CD4 count was not used for these analyses due to high number of missing values. Differences in survival time between groups of subjects by age categories and by sex were investigated using Kaplan–Meier survival plots. Data analysis was performed using SAS v.9.2.

For the Cox proportional hazards model and Kaplan-Meier survival curves, analyses excluded those whose follow-up status was characterized in the dataset as “missing” or suspected of having “defaulted.” This represented 22.7% (N = 2475) of all those aged 25 and older who had initiated in the five year period from 1 July 2005 to 30 June 2010. Based on a systematic review [24] and data from two studies in Malawi, [25], [26] we estimate that approximately 41–50% of those lost to follow-up would have died within approximately two years. Analyses were run to identify differences in characteristics between those in the analyzed cohort versus those excluded.

### Ethics Statement

The study analysis received approval from the National Health Sciences Research Committee of Malawi in April 2011.

## Results

There were 10888 patients aged 25 and older who had initiated ART between 1 July 2005 and 30 June 2010 in the database. Of those, 6789 (62.4%) were female; 9469 (87.0%) were aged 25–49, 1074 (9.9%) were 50–59 and 345 (3.2%) were aged 60 and older. The older age groups had significantly (P<0.0001) higher percentages of male patients than the younger age groups: 44.9% of those 50 years and older were male compared to 36.6% of those aged under 50 years (table 1). Older adults were also more likely to be living in a rural location compared to younger adults (P = 0.003). There were no significant differences between age groups for year of initiation or TB status at baseline.

**Table 1:** Characteristics of patients at initiation of antiretroviral therapy.

		25–49		50–59		60+		
		N	%	N	%	N	%	P Value (25–49 vs 50+)
Sex								
	Female	6007	63.4%	613	57.1%	169	49.0%	<0.0001
	Male	3462	36.6%	461	42.9%	176	51.0%	
WHO Stage / Reason for ART Initiation								
	Stage 1	341	3.6%	29	2.7%	10	2.9%	0.188
	Stage 2	3300	34.9%	363	33.9%	121	35.4%	
	Stage 3	4281	45.3%	511	47.8%	177	51.8%	
	Stage 4	1530	16.2%	167	15.6%	34	9.9%	
	Missing^a^	17	*0.2%*	3	*0.3%*	3	*0.9%*	
ART Location								
	Urban	4971	52.5%	525	48.9%	154	44.6%	0.003
	Semi Urban	1600	16.9%	177	16.5%	71	20.6%	
	Rural	2898	30.6%	372	34.6%	120	34.8%	
CD4 cell counts at baseline (cells/µl)								
	less than or equal to 50	608	13.7%	39	7.7%	10	5.9%	0.001
	between 50 and 99	748	16.8%	87	17.2%	28	16.6%	
	between 100 and 199	1515	34.0%	179	35.4%	55	32.5%	
	between 200 and 299	1388	31.2%	184	36.4%	68	40.2%	
	300 or over	192	4.3%	17	3.4%	8	4.7%	
	Missing^a^	5018	*53.0%*	569	*52.9%*	176	*51.0%*	
	Median baseline CD4	157		171		185		
Tuberculosis status at baseline								
	TB past or present	1406	14.8%	155	14.4%	52	15.1%	0.797
	No TB	8063	85.2%	919	85.6%	293	84.9%	
Year of ART Initiation								
	2009–2010	3827	40.4%	415	38.6%	150	43.5%	0.287
	2007–2008	4354	46.0%	525	48.9%	154	44.6%	
	2005–2006	1287	13.6%	134	12.5%	41	11.9%	

a Missing values not included in percentages in categories above.

Zomba District by age category 25–49,50–59 and 60 and older who initiated treatment from July 2005 to June 2010.

After adjusting for location and sex, age group was not associated with WHO clinical stage at initiation of ART or with the number of HIV-related conditions at initiation. Females were slightly more likely than males to have two or more HIV-related conditions at initiation (odds ratio (OR): 1.17 (95% confidence interval (CI): 1.05 to 1.32)). Those who initiated in semi-urban and rural areas were less likely (OR: 0.61 (95% CI: 0.53 to 0.69)) to have two or more conditions compared to urban patients. Interactions between age and location and between age and sex were tested and were not significant.

**Figure 1:**
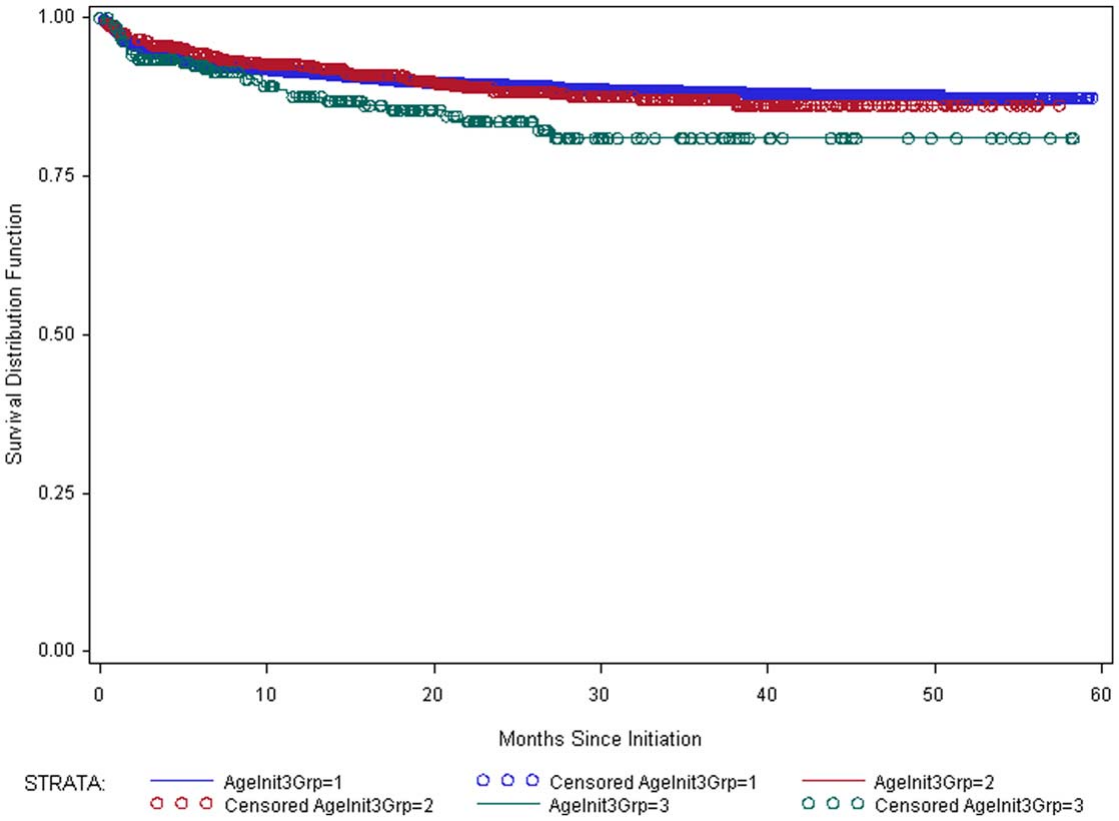
Survival estimates by age. Kaplan-Meier survival estimates by age group for those aged 25 and older who initiated treatment in Zomba District between 1 July 2005 and 30 June 2010. Blue is 25–49 (N = 7297); Red is 50–59 (N = 839); Green is 60+ (N = 276).

**Figure 2:**
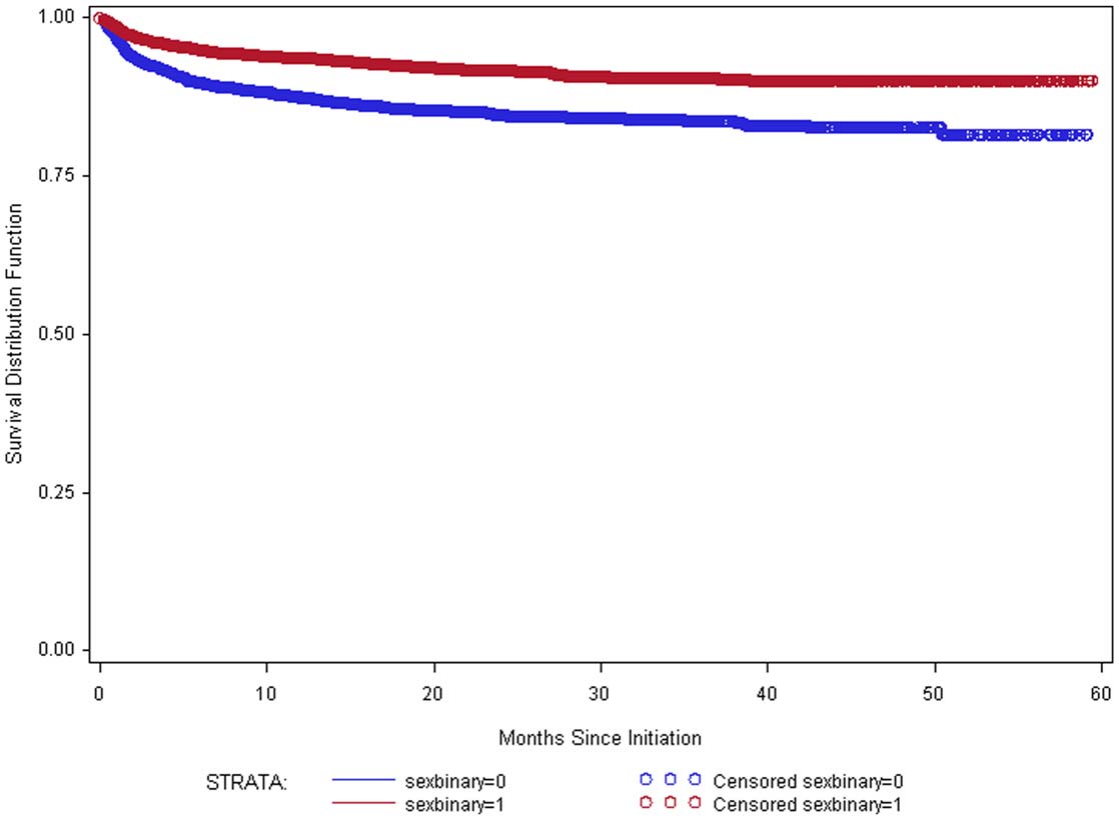
Survival estimates by sex. Kaplan-Meier survival estimates by sex for those aged 25 and older who initiated treatment in Zomba District between 1 July 2005 and 30 June 2010. Blue is Male (N = 3057); Red is Female (N = 5355).

Data were available for 8412 individuals for the analysis of treatment outcomes. Of these, 765 (9.1%) died within 5 years of initiation of treatment. Survival rates among those aged 50–59 were not statistically different from those aged 25–49 (P = 0.92) (figure 1). Survival among those aged 60 and older was significantly worse (P = 0.02) than among those 25–59. Those aged 60 and older also had significantly worse survival (P = 0.04) than those aged 50–59. Further analysis revealed that sex was a significant predictor of survival (P<0.0001), with males having worse survival than females (figure 2).

After controlling for sex and WHO clinical stage at initiation, the hazard ratio (HR) for those aged 50–59 compared to those aged 25–49 was 1.00 (95% CI: 0.79 to 1.27; P = 0.99) (table 2). Compared to those aged 25–49 years, those aged 60 and older were at significant increased risk of dying (HR = 1.46 (95% CI: 1.03 to 2.06; P = 0.03)). Controlling for stage and age group, being female reduced the risk of death with a hazard ratio of 0.58 (95% CI: 0.50 to 0.67; P<0.0001). As expected, WHO clinical stage was strongly associated with mortality. After controlling for sex and age group, compared to Stage 1, the hazard ratio for Stage 3 was 5.4 (95% CI: 1.74 to 16.94) and the hazard ratio for Stage 4 was 12.3 (95% CI: 3.93 to 38.32).

**Table 2:** Cox proportional hazards models for mortality.

		Model with age, sex and WHO Stage at initiation (N = 8390)			Model with Age and Sex (N = 8412)		
Variable		Hazard ratio	95% confidence limits	P Value	Hazard ratio	95% confidence limits	P Value
Age group				0.098			0.216
	25–49	1.00			1.00		
	50–59	1.00	0.79–1.27	0.998	0.97	0.77–1.23	0.826
	60+	1.46	1.03–2.06	0.032	1.35	0.96–1.91	0.086
Sex		0.58	0.50–0.67	<0.0001	0.52	0.46–0.60	<0.0001
WHO Stage				<0.0001			
	Stage 1	1.00					
	Stage 2	2.38	0.76–7.49	0.139			
	Stage 3	5.43	1.74–16.94	0.004			
	Stage 4	12.27	3.93–38.32	<0.0001			

Of the 765 who died, 629 (82.2%) died within the first year of ART initiation. The pattern of mortality between the age groups was unchanged with those 50–59 have similar mortality to those aged 25–49 with those 60 and older having higher mortality.

Due to missing outcome data, 2475 (22.7%) of subjects were excluded from survival analyses. The mean age and baseline CD4 of those included in analysis were not meaningfully different from the mean age of those excluded but those excluded were more likely to be male and were more likely to have been characterized as WHO Stage 4 on initiation of treatment (table 3). Importantly, the relative differences in sex and WHO Stage between those included and those excluded from survival analyses were largely consistent among those over 50 and among those under 50.

**Table 3:** Comparison of those included in survival analysis and those excluded.

		Included in Analysis (N)	Excluded from Analysis (N)
Total population	Age (mean)	38.3 (8412)	38.0 (2475)
	Sex (% male)	36.3% (3057)	42.1% (1042)
	WHO Stage 3 and 4 (%)	61.1% (5122)	63.8 (1578)
	Baseline CD4 (mean)	162.6 (4055)	154.6 (1070)
25 to 49	Age (mean)	35.5 (7297)	35.5 (2171)
	Sex (% male)	35.2% (2569)	41.1% (893)
	WHO Stage 3 and 4 (%)	60.8% (4425)	63.9% (1386)
50 and older	Age (mean)	56.1 (1115)	56.0 (304)
	Sex (% male)	43.8% (488)	49.0% (149)
	WHO Stage 3 and 4 (%)	62.9% (697)	63.2% (192)

If the excluded patients are included in the survival analysis up to the point when they were classified as missing, the results and significance noted above remained in the same direction and changed only slightly in magnitude. The hazard ratio for those aged 60 and older became 1.52 (95% CI: 1.08 to 2.15; P = 0.0168) while the hazard ratio for those aged 50–59 remained 1.00 compared to those aged 25–49. Using the Kaplan-Meier survival curves, when including those with incomplete outcome data, those 60 and older had significantly worse survival than those 50–59 (P = 0.03).

## Discussion

Adults aged 50 and older in Zomba District, Malawi, who initiated ART were more likely than younger adults to be male and be receiving treatment at a rural health facility. However, based on WHO clinical stage and number of HIV-related conditions at ART initiation, older adults in this population were no sicker at presentation than younger adults.

We found no difference in mortality on treatment among those aged 50 to 59 years compared to those aged 25 to 49, indicating that people in their fifties respond just as well to treatment as younger people. Survival among those aged 60 and older was reduced; however, this is to be expected due to higher rates of all-cause mortality among older adults. In this study, sex was a much better predictor of survival than age in the Zomba District ART cohort. This study suggests that females are more likely to survive than men.

In our study, 9% of those initiated on treatment died over the course of the five years. This level of mortality was higher than was found in Uganda [16] but comparable to data seen in South Africa, Cote d'Ivoire and elsewhere in Malawi [15].

The lack of significant differences in the clinical condition of older adults at initiation of ART is contrary to what has been hypothesized. In Brazil, those aged 60 and older had more AIDS-defining diseases at diagnosis than those aged 20–39 [8] and a review of developed country studies found that older adults presented later and sicker [6]. However, the recent South African ART among older adults study found that they did not present with more advanced disease than younger individuals [17]. Similar to our findings, the Ugandan study by Bakanda and colleagues also found that older adults were more likely to be male and did not differ in WHO clinical stage at treatment initiation [16].

With regard to treatment outcomes, the Ugandan study found that being aged 50 and older was associated with mortality compared to those 18 to 49 after controlling for other variables [16]. The South African study found that older adults had 32% excess mortality compared to those 25 to 49 [17]. A four site cross-Africa study found that treatment outcomes for over 50s were worse than for younger age groups [15]. Ours is the first study that breaks down the over 50 group into additional age categories for analysis.

Especially for the 50–59 age group, our data confirms what has been seen in non-African studies where older HIV-infected patients responded as well to ART as younger age groups [27]–[30]. A Brazilian study that compared those aged 20–39 against those aged 60 and older found that, once on ART, mortality between the two groups was similar [8]. A recent case-control study, in which the outcome in patients aged 55 years or older was compared with patients aged 35 years or younger, all treated with ART, concluded that the virological outcome did not differ between groups [31]. The picture is complex however as a number of other studies have found that there is slower recovery of CD4 levels among older adults [12], [32]. The eventual higher mortality among older adults in the study was expected and has been seen in other studies [33], [34].

Our finding that women on ART are more likely to survive than men has been observed in other studies in Malawi [35] and across Africa [36]. Potential reasons for this sex difference could be that men seek care at a more advanced stage of illness and have poorer levels of adherence.

The strength of the study is its inclusion of program data from an entire district's ART population including urban and rural sites. However, study limitations include concerns regarding accuracy and consistency that are associated with use of operational data. Because data were extracted from routine monitoring and evaluation indicators, information that might be included in a prospective study is not available, as it is not a part of routine clinical care for ART provision under the Malawi MOH public health model and is reflective of resource limitations in that setting. Specifically, high levels of missing baseline CD4 data made usage of that variable difficult.

A further limitation was the approximately 20% loss to follow up in the dataset. However, the levels of loss to follow-up in this study are comparable to those seen in a review of loss to follow-up across Africa [24]. The exclusion of those lost to follow-up from analysis is likely to have led to underestimation of the absolute number of deaths and mortality rates. However, because the magnitude of differences in characteristics of those included and excluded was similar in the different age groups, the relative risk of death (or hazard ratio) comparing those aged 50 years and over with those aged under 50 was unlikely to be biased.

While some studies have also excluded those lost to follow-up, [15] other studies have used randomization techniques to impute outcome status [16], [37]. These imputation techniques make assumptions about which variables are correlated with mortality. For example, Bakanda and colleagues [16] assumed that those with lower CD4 count and those who were older were more likely to die. We did not conduct outcome imputation for those lost to follow-up. Given that age and its relation to mortality was the primary outcome of this study, it was felt that including age in the imputation method would be inappropriate as the method of imputation would be directly related to the primary outcome of interest. Baseline CD4 was also not ideal for use as an imputation variable due to the large number of non-random missing values in our dataset. In the future, greater efforts will be needed to trace those lost to follow-up and examine specific characteristics and determinants of mortality [38], [39].

That older adults respond well to ART despite a lack of specifically targeted services is an encouraging sign for ART scale-up. This evidence can be used to combat ageist stigma [40] and to encourage promotion of services among this age group. There have been suggestions there might be higher adherence rates among the elderly – which has been found in developed countries [27], [41] – which may be driving the positive treatment response.

With the ageing of the epidemic, HIV has become a complex chronic disease characterised by increasing rates of co-morbid conditions including liver and renal disease, cancers, osteoporosis, neurocognitive and cardiovascular diseases [5]. It is well known that older people's immune systems weaken with age regardless of HIV status leaving older people at greater susceptibility to a range of conditions [42], [43] – but this has not been specifically studied in Africa among those on ART. The ageing of the epidemic has implications on service delivery with a growing need for integrated diagnostic and treatment services for HIV and non-communicable diseases.

Older adults have been largely neglected by the global HIV response but there are encouraging signs that those aged 50 and older are starting to be seen by the HIV community as productive, fully active contributors, care givers, and educators, whose susceptibility to HIV needs to be integrated into the core of the response to the epidemic.
